# 7-Chloro-5-methyl-2-phenyl­pyrazolo­[1,5-*a*]pyrimidine

**DOI:** 10.1107/S1600536813009896

**Published:** 2013-04-17

**Authors:** Ibtissam Bassoude, Sabine Berteina-Raboin, El Mokhtar Essassi, Gérald Guillaumet, Lahcen El Ammari

**Affiliations:** a Laboratoire de Chimie Organique Hétérocyclique URAC21, Pôle de Compétences Pharmacochimie, Université Mohammed V-Agdal, Avenue Ibn Battouta, BP 1014, Rabat, Morocco; b Institut de Chimie Organique et Analytique, Université d’Orléans, UMR CNRS 6005, BP 6759, 45067 Orléans Cedex 2, France; c Institute of Nanmaterials and Nanotechnology, MASCIR, Rabat, Morocco; d Laboratoire de Chimie du Solide Appliquée, Université Mohammed V-Agdal, Faculté des Sciences, Avenue Ibn Battouta, BP 1014, Rabat, Morocco

## Abstract

The fused pyrazole and pyrimidine rings in the title compound, C_13_H_10_ClN_3_, are almost coplanar, their planes being inclined to one another by 0.8 (2)°. The mean plane of the fused ring system is nearly coplanar with the phenyl ring, as indicated by the dihedral angle between their planes of 9.06 (7)°.

## Related literature

For pharmacological and biochemical properties of pyra­zolo­[1,5-*a*]pyrimidine derivatives, see: Selleri *et al.* (2005 ▶); Almansa *et al.* (2001 ▶); Suzuki *et al.* (2001 ▶), Chen *et al.* (2004 ▶). For related structures, see: Senga *et al.* (1981 ▶).
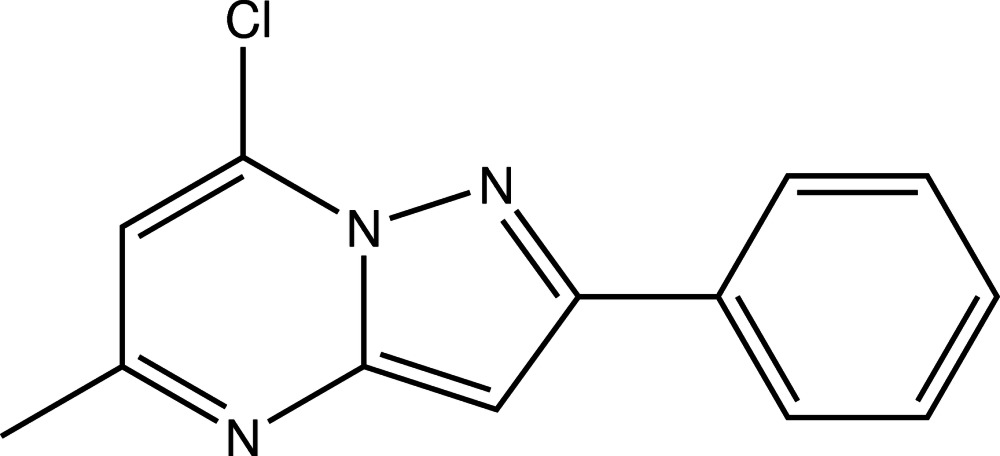


## Experimental

### 

#### Crystal data

C_13_H_10_ClN_3_
*M* *_r_* = 243.69
Monoclinic, 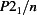

*a* = 6.5993 (2) Å
*b* = 12.6166 (4) Å
*c* = 13.8702 (5) Å
β = 100.131 (2)°
*V* = 1136.84 (6) Å^3^
*Z* = 4
Mo *K*α radiation
μ = 0.31 mm^−1^
*T* = 296 K
0.41 × 0.32 × 0.21 mm

#### Data collection

Bruker X8 APEXII area-detector diffractometer
16957 measured reflections
2925 independent reflections
2521 reflections with *I* > 2σ(*I*)
*R*_int_ = 0.024

#### Refinement

*R*[*F* ^2^ > 2σ(*F* ^2^)] = 0.035
*wR*(*F* ^2^) = 0.103
*S* = 1.06
2925 reflections
154 parameters
H-atom parameters constrained
Δρ_max_ = 0.23 e Å^−3^
Δρ_min_ = −0.23 e Å^−3^

### 

Data collection: *APEX2* (Bruker, 2009 ▶); cell refinement: *SAINT* (Bruker, 2009 ▶); data reduction: *SAINT*; program(s) used to solve structure: *SHELXS97* (Sheldrick, 2008 ▶); program(s) used to refine structure: *SHELXL97* (Sheldrick, 2008 ▶); molecular graphics: *ORTEP-3 for Windows* (Farrugia, 2012 ▶); software used to prepare material for publication: *PLATON* (Spek, 2009 ▶) and *publCIF* (Westrip, 2010 ▶).

## Supplementary Material

Crystal structure: contains datablock(s) I, global. DOI: 10.1107/S1600536813009896/rz5056sup1.cif

Structure factors: contains datablock(s) I. DOI: 10.1107/S1600536813009896/rz5056Isup2.hkl

Supplementary material file. DOI: 10.1107/S1600536813009896/rz5056Isup3.cml

Additional supplementary materials:  crystallographic information; 3D view; checkCIF report

## Figures and Tables

**Fig. 1.**
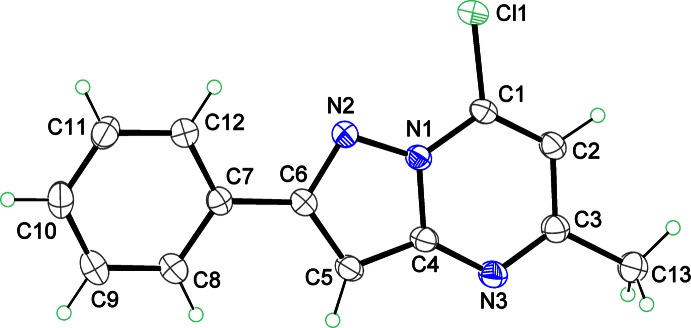
The molecular structure of the title compound with displacement ellipsoids drawn at the 50% probability level. H atoms are represented as small circles of arbitrary radius.

## supplementary crystallographic information

### Comment

Pyrazolo[1,5-*a*]pyrimidines have attracted considerable interest because of their biological activity. For instance, they are known for their potent utility as selective peripheral benzodiazepine receptor ligands (Selleri *et al.*, 2005), COX-2 selective inhibitors (Almansa *et al.*, 2001), HMG-CoA reductase inhibitors (Suzuki *et al.*, 2001) and CRF1 antagonists (Chen *et al.*, 2004). Our research group targeted at the synthesis of heterocycles with a bridgehead nitrogen atom such as the title compound (Senga *et al.* 1981).

The crystal structure of the title compound is built up from two fused five and six-membered rings (N1/N2/C4–C6 and N1/N3/C1–C4) linked to a methyl group and to a phenyl ring (C7–C12) as shown in Fig. 1. The pyrazole and pyrimidine rings are essentially planar with maximum deviations of 0.0010 (13) Å and 0.0052 (13) Å for C6 and C1, respectively, and form a dihedral angle of 0.8 (2)°. The mean plane through the fused ring system makes a dihedral angle of 9.06 (7)° with the phenyl ring.

### Experimental

Under argon, a mixture of 7-hydroxy-5-methyl-2-phenylpyrazolo[1,5-*a*]pyrimidine (0.8 g, 3.5 mmol), phosphorus oxychloride (0.8 ml, 8.89 mmol) and triethylamine (1 mL, 7 mmol) in 1,4-dioxane (2 ml) was heated to reflux for 3 h. The reaction mixture was allowed to cool to room temperature. After evaporation of the solvent under reduced pressure and addition of a NaHCO_3_ saturated solution at 273 K (pH = 8), the residue was extracted with CH_2_Cl_2_. The combined organic layers were dried with MgSO_4_, concentrated under vacuum and the residue was purified on silica gel by column chromatography using a 9:1 (*v*/*v*) mixture of petroleum ether and ethyl acetate as eluent. The compound was recrystallized from a mixture of cyclohexane/CH_2_Cl_2_ (1:1 *v*/*v*) to give colourless crystals.

### Refinement

All H atoms could be located in a difference Fourier map and treated as riding with C—H = 0.93 Å (aromatic), and C—H = 0.96 Å (methyl) and with *U*_iso_(H) = 1.2 *U*_eq_(aromatic) or *U*_iso_(H) = 1.5 *U*_eq_ (methyl).

### Crystal data

**Table:**

C_13_H_10_ClN_3_	*F*(000) = 504
*M**_r_* = 243.69	*D*_x_ = 1.418 Mg m^−^^3^
Monoclinic, *P*2_1_/*n*	Mo *K*α radiation, λ = 0.71073 Å
Hall symbol: -P 2yn	Cell parameters from 2925 reflections
*a* = 6.5993 (2) Å	θ = 3.0–28.7°
*b* = 12.6166 (4) Å	µ = 0.31 mm^−^^1^
*c* = 13.8702 (5) Å	*T* = 296 K
β = 100.131 (2)°	Block, colourless
*V* = 1136.84 (6) Å^3^	0.41 × 0.32 × 0.21 mm
*Z* = 4	

### Data collection

**Table:**

Bruker X8 APEXII area-detector diffractometer	2521 reflections with *I* > 2σ(*I*)
Radiation source: fine-focus sealed tube	*R*_int_ = 0.024
Graphite monochromator	θ_max_ = 28.7°, θ_min_ = 3.0°
φ and ω scans	*h* = −6→8
16957 measured reflections	*k* = −17→17
2925 independent reflections	*l* = −18→18

### Refinement

**Table:**

Refinement on *F*^2^	Primary atom site location: structure-invariant direct methods
Least-squares matrix: full	Secondary atom site location: difference Fourier map
*R*[*F*^2^ > 2σ(*F*^2^)] = 0.035	Hydrogen site location: inferred from neighbouring sites
*wR*(*F*^2^) = 0.103	H-atom parameters constrained
*S* = 1.06	*w* = 1/[σ^2^(*F*_o_^2^) + (0.0523*P*)^2^ + 0.2738*P*] where *P* = (*F*_o_^2^ + 2*F*_c_^2^)/3
2925 reflections	(Δ/σ)_max_ = 0.001
154 parameters	Δρ_max_ = 0.23 e Å^−^^3^
0 restraints	Δρ_min_ = −0.23 e Å^−^^3^

### Special details

**Table:**

Geometry. All e.s.d.'s (except the e.s.d. in the dihedral angle between two l.s. planes) are estimated using the full covariance matrix. The cell e.s.d.'s are taken into account individually in the estimation of e.s.d.'s in distances, angles and torsion angles; correlations between e.s.d.'s in cell parameters are only used when they are defined by crystal symmetry. An approximate (isotropic) treatment of cell e.s.d.'s is used for estimating e.s.d.'s involving l.s. planes.
Refinement. Refinement of *F*^2^ against all reflections. The weighted *R*-factor *wR* and goodness of fit *S* are based on *F*^2^, conventional *R*-factors *R* are based on *F*, with *F* set to zero for negative *F*^2^. The threshold expression of *F*^2^ > σ(*F*^2^) is used only for calculating *R*-factors(gt) *etc*. and is not relevant to the choice of reflections for refinement. *R*-factors based on *F*^2^ are statistically about twice as large as those based on *F*, and *R*- factors based on all data will be even larger.

### Fractional atomic coordinates and isotropic or equivalent isotropic displacement parameters (Å^2^)

**Table:**

	*x*	*y*	*z*	*U*_iso_*/*U*_eq_	
C1	0.07651 (19)	0.69856 (10)	0.25074 (9)	0.0344 (3)	
C2	−0.0283 (2)	0.72024 (10)	0.32394 (10)	0.0393 (3)	
H2	−0.1459	0.7622	0.3121	0.047*	
C3	0.0426 (2)	0.67836 (11)	0.41889 (10)	0.0420 (3)	
C4	0.3143 (2)	0.59912 (10)	0.36620 (9)	0.0369 (3)	
C5	0.4894 (2)	0.54075 (11)	0.36126 (9)	0.0398 (3)	
H5	0.5708	0.5041	0.4120	0.048*	
C6	0.51851 (19)	0.54836 (10)	0.26417 (9)	0.0349 (3)	
C7	0.68242 (19)	0.49912 (10)	0.21982 (9)	0.0353 (3)	
C8	0.8475 (2)	0.44870 (11)	0.27861 (11)	0.0423 (3)	
H8	0.8546	0.4468	0.3462	0.051*	
C9	1.0010 (2)	0.40136 (12)	0.23699 (13)	0.0502 (4)	
H9	1.1112	0.3684	0.2768	0.060*	
C10	0.9911 (2)	0.40282 (13)	0.13685 (13)	0.0522 (4)	
H10	1.0940	0.3706	0.1092	0.063*	
C11	0.8287 (2)	0.45210 (13)	0.07785 (12)	0.0521 (4)	
H11	0.8215	0.4528	0.0103	0.063*	
C12	0.6755 (2)	0.50072 (12)	0.11910 (11)	0.0448 (3)	
H12	0.5671	0.5347	0.0789	0.054*	
C13	−0.0778 (3)	0.69939 (16)	0.49944 (12)	0.0599 (4)	
H13A	−0.1946	0.7430	0.4748	0.090*	
H13B	0.0084	0.7352	0.5525	0.090*	
H13C	−0.1239	0.6334	0.5224	0.090*	
N1	0.24981 (16)	0.63830 (8)	0.27118 (7)	0.0332 (2)	
N2	0.37353 (16)	0.60790 (9)	0.20808 (8)	0.0357 (2)	
N3	0.20986 (19)	0.61941 (10)	0.43971 (8)	0.0432 (3)	
Cl1	0.00409 (5)	0.74223 (3)	0.13336 (2)	0.04662 (13)	

### Atomic displacement parameters (Å^2^)

**Table:**

	*U*^11^	*U*^22^	*U*^33^	*U*^12^	*U*^13^	*U*^23^
C1	0.0348 (6)	0.0302 (5)	0.0367 (6)	0.0020 (5)	0.0021 (5)	0.0041 (5)
C2	0.0389 (7)	0.0361 (6)	0.0427 (7)	0.0089 (5)	0.0061 (5)	0.0022 (5)
C3	0.0462 (7)	0.0413 (7)	0.0385 (6)	0.0091 (6)	0.0078 (5)	−0.0005 (5)
C4	0.0393 (6)	0.0370 (6)	0.0326 (6)	0.0047 (5)	0.0014 (5)	0.0011 (5)
C5	0.0401 (7)	0.0423 (7)	0.0351 (6)	0.0103 (5)	0.0012 (5)	0.0014 (5)
C6	0.0331 (6)	0.0323 (6)	0.0382 (6)	0.0011 (5)	0.0029 (5)	−0.0004 (5)
C7	0.0318 (6)	0.0319 (6)	0.0421 (6)	−0.0016 (5)	0.0061 (5)	−0.0006 (5)
C8	0.0379 (7)	0.0412 (7)	0.0459 (7)	0.0033 (5)	0.0021 (5)	−0.0029 (5)
C9	0.0359 (7)	0.0483 (8)	0.0647 (10)	0.0074 (6)	0.0044 (6)	−0.0021 (7)
C10	0.0412 (8)	0.0508 (8)	0.0691 (10)	0.0031 (6)	0.0219 (7)	−0.0026 (7)
C11	0.0527 (8)	0.0576 (9)	0.0508 (8)	0.0019 (7)	0.0224 (7)	0.0033 (7)
C12	0.0414 (7)	0.0502 (8)	0.0436 (7)	0.0053 (6)	0.0095 (6)	0.0043 (6)
C13	0.0659 (10)	0.0735 (11)	0.0433 (8)	0.0281 (9)	0.0176 (7)	0.0038 (7)
N1	0.0334 (5)	0.0318 (5)	0.0338 (5)	0.0036 (4)	0.0042 (4)	0.0023 (4)
N2	0.0344 (5)	0.0360 (5)	0.0368 (5)	0.0026 (4)	0.0068 (4)	0.0024 (4)
N3	0.0479 (6)	0.0474 (6)	0.0335 (5)	0.0130 (5)	0.0053 (5)	0.0004 (5)
Cl1	0.0463 (2)	0.0521 (2)	0.04017 (19)	0.00949 (14)	0.00399 (14)	0.01307 (13)

### Geometric parameters (Å, º)

**Table:**

C1—C2	1.3533 (18)	C7—C8	1.3940 (18)
C1—N1	1.3610 (16)	C8—C9	1.386 (2)
C1—Cl1	1.7055 (13)	C8—H8	0.9300
C2—C3	1.4197 (19)	C9—C10	1.379 (2)
C2—H2	0.9300	C9—H9	0.9300
C3—N3	1.3201 (18)	C10—C11	1.377 (2)
C3—C13	1.504 (2)	C10—H10	0.9300
C4—N3	1.3519 (17)	C11—C12	1.389 (2)
C4—C5	1.3818 (18)	C11—H11	0.9300
C4—N1	1.4019 (16)	C12—H12	0.9300
C5—C6	1.3966 (18)	C13—H13A	0.9600
C5—H5	0.9300	C13—H13B	0.9600
C6—N2	1.3503 (16)	C13—H13C	0.9600
C6—C7	1.4727 (17)	N1—N2	1.3532 (15)
C7—C12	1.3897 (19)		
			
C2—C1—N1	118.58 (11)	C10—C9—C8	120.34 (14)
C2—C1—Cl1	123.81 (10)	C10—C9—H9	119.8
N1—C1—Cl1	117.61 (9)	C8—C9—H9	119.8
C1—C2—C3	119.48 (12)	C11—C10—C9	119.88 (14)
C1—C2—H2	120.3	C11—C10—H10	120.1
C3—C2—H2	120.3	C9—C10—H10	120.1
N3—C3—C2	122.62 (12)	C10—C11—C12	120.10 (15)
N3—C3—C13	117.87 (12)	C10—C11—H11	119.9
C2—C3—C13	119.50 (13)	C12—C11—H11	119.9
N3—C4—C5	132.85 (12)	C11—C12—C7	120.65 (14)
N3—C4—N1	122.10 (11)	C11—C12—H12	119.7
C5—C4—N1	105.05 (11)	C7—C12—H12	119.7
C4—C5—C6	105.62 (11)	C3—C13—H13A	109.5
C4—C5—H5	127.2	C3—C13—H13B	109.5
C6—C5—H5	127.2	H13A—C13—H13B	109.5
N2—C6—C5	112.96 (11)	C3—C13—H13C	109.5
N2—C6—C7	119.48 (11)	H13A—C13—H13C	109.5
C5—C6—C7	127.56 (11)	H13B—C13—H13C	109.5
C12—C7—C8	118.62 (12)	N2—N1—C1	127.17 (10)
C12—C7—C6	121.13 (12)	N2—N1—C4	112.96 (10)
C8—C7—C6	120.25 (12)	C1—N1—C4	119.86 (11)
C9—C8—C7	120.39 (14)	C6—N2—N1	103.41 (10)
C9—C8—H8	119.8	C3—N3—C4	117.35 (11)
C7—C8—H8	119.8		
